# Factors Associated With Self-Reported Practices on Food Safety Among Female Food Handlers of Households in Budhiganga Rural Municipality, Nepal

**DOI:** 10.1155/bmri/3233795

**Published:** 2025-08-08

**Authors:** Jibesh Acharya, Pravat Uprety

**Affiliations:** Central Department of Statistics, Institute of Science and Technology, Tribhuvan University, Kathmandu, Nepal

**Keywords:** food, food handlers, Nepal, safety, self-reported practices

## Abstract

This study examines the level of self-reported food safety practices and the factors associated with them. Using a structured questionnaire, this study was conducted among 335 female household food handlers in the Budhiganga Rural Municipality, Nepal. The overall level of the practice was assessed through 14 questions on a 3-point scale (1 = *always*, 2 = *sometimes*, and 3 = *never*) where the mean cumulative score was taken as the cut-off value. Over half (57.30%) of food handlers showed good safety practices. Education—no education (AOR = 3.01) and primary education (AOR = 2.50), ethnicity—Madhesi/Dalit/Muslim (AOR = 2.43), awareness about food safety (AOR = 2.48), ownership of water sources (AOR = 1.88), fuel used for cooking—wood (AOR = 0.25), water facility inside the toilet (AOR = 1.94), and knowledge of food safety (AOR = 4.40) were the significant factors associated with different levels of practice. The study highlighted the importance of education, accessibility, and availability of water sources in households to minimize risks associated with foodborne diseases in Nepal.

## 1. Background

Food safety is the correct handling, arranging, and conserving of food, which prevents contamination and the spread of illnesses from food consumption [1]. It is regarded as a method focused on safeguarding public well-being and forestalling illness caused by ingesting contaminated or mishandled food [2]. In general, to ensure food safety, the United Nations has put forward five primary principles: using safe water and raw materials, cooking food thoroughly, separating raw and cooked foods, maintaining hygiene, and storing food at the proper temperature [3].

Foodborne illnesses arise when consuming food or beverages with harmful microorganisms and have sporadic breakouts in the least developed and developing nations [4]. Due to the lack of food safety awareness, compromised hygiene, low living standards, limited access to health care, and so forth, economic prosperity is challenging [2, 5, 6]. Thus, assuring food safety in a country is crucial since it enhances agricultural production, increases market accessibility, promotes tourism, and ultimately supports sustainable development [7]. According to WHO [8], globally, 31 foodborne hazards causing 32 diseases resulted in around 600 million foodborne illnesses (one in every 10 people) and 420,000 deaths. Consequently, though foodborne diseases are more frequent, they are preventable, but underreporting the instances of these diseases has been an increasing burden on public health [9, 10].

Food contamination is directly linked to the health status of food handlers, their hygiene practices, and their understanding and adherence to food safety regulations [11, 12]. Though food handlers have a vital role in maintaining food hygiene, approximately 10%–20% of foodborne diseases are due to food contamination by food handlers. Also, a huge number of household food handlers are not well informed about food safety procedures for minimizing health risks during preparation in the home [9, 13]. In addition to this, studies [13–16] have shown that the rise of foodborne diseases often occurs in the home. The incidences of foodborne diseases due to handling errors are much higher, and as a result of their sporadic and mild nature, they remain underreported [9, 16–18].

In domestic kitchens, foodborne illnesses result from practices like rushing in food preparation, inadequately reheating the leftovers, poor sanitation, permitting infected individuals in food handling, using ingredients from unknown sources, and so forth [14, 19]. It is estimated that a significant percentage, 50%–87%, of reported foodborne illness outbreaks are the result of food prepared at home [20].

In Nepal, it is a common practice for people to have their meals at home, and it is expected that a woman be the food handler. So, these female food handlers play a pivotal role in every stage of food preparation, the prevention of foodborne diseases, and the assurance of food safety at home. Since they are the final safeguard of food in the food safety chain [18, 21], they should be aware of proper food handling methods and at least bear the fundamental knowledge required to keep themselves and their families safe from foodborne diseases. In the context of Nepal, foodborne diseases are one of the main reasons for both morbidity and mortality [22, 23]; hence, it is important to understand the practices of food safety adopted in households. Also, due to scarce studies about food safety practices in households in Nepal, this study holds significant importance in shaping national policies accordingly.

## 2. Methods

### 2.1. Study Design

A household cross-sectional study design was used in this study. Out of seven wards in Budhiganga Rural Municipality, Ward Number 4 was selected purposively. In Nepalese households, generally, mothers/women are involved in preparing food for their children and family members. In a similar context regarding Budhiganga Rural Municipality, the primary female food handler of the respective household was taken as the study unit. In the cases of households having more than one female food handler, only one of them was selected randomly.

### 2.2. Questionnaire Design

The questionnaire employed in the study comprised three sections, namely, the sociodemographic section (11 questions), the knowledge section (16 questions), and the practice section (14 questions). The knowledge and practice sections were developed based on different published literature based on the assessment of food handler's knowledge and practice of food safety [1, 14, 24–29]. Minor modifications of some questions were done concerning food-related practices in Nepal.

### 2.3. Data Collection

As per the record of Budhiganga Rural Municipality, there were 1265 households in Ward Number 4. Thus, for this population, the sample size was determined by using Yamane's formula [30] at a 5% margin of error. The sample size (*n*) was as follows:

 $$\begin{array}{c} n=N/{\left(1+N*e\right)}^{2}=1265/{\left(1+1265*0.05\right)}^{2}=303.90\approx 304. \end{array}$$

And, taking a 10% nonresponse rate, the final sample size for the study was 335.

Households were selected using a simple random sampling method. The data was collected from the food handlers by going to their households. The face-to-face interview method was used, and the researcher filled out the questionnaire. The questionnaire was filled out in about 20 min.

### 2.4. Data Analysis

At first, the obtained information was entered into Microsoft Excel, and the R software was used to perform all statistical analyses. Both descriptive (frequency and percentage) and inferential (chi-square test and multiple binary logistic regression) statistical methods were used. Model adequacy tests like the Hosmer–Lemeshow test and Omnibus Chi-square test were used.

For knowledge, 16 questions with three responses; “Yes”, “No”, and “I don't know” were asked. Respondents got 1 score for the correct answer and 0 for the incorrect one. On the cumulative sum of the scores obtained from 16 questions, less than 70% of the cumulative score indicated having “poor knowledge” and greater than or equal to 70% of the cumulative score indicated having “adequate knowledge” of food safety [1, 27].

For assessing the food safety practices, 14 questions with a 3-point scale (1 = *always*, 2 = *sometimes*, and 3 = *never*) were asked, where respondents posed “good practice” if the cumulative score obtained was greater than or equal to the mean score, otherwise, “bad practice” [2]. The reverse order of scoring was adopted for negatively framed questions.

## 3. Results

### 3.1. Profile of the Participants

A total of 335 primary food handlers were interviewed in the study. The majority of the respondents were aged 25–44 years. In terms of educational background, 52 (15.50%) had no formal education. Most of the participants (42.10%) belonged to the Brahmin/Chhetri ethnic group, followed by participants from Madheshi/Dalit/Muslim ethnic groups (34.00%). Most of the participants had less than NRs. 20,000 monthly income in their household. Regarding the family structure of the participants, the majority (73.70%) had less than five members in their household, while 77.30% had children under 15 years of age. Among the participants, only 40.60% had heard/knew about food safety through different sources. Regarding environment-related factors, 39 (11.60%) of the respondents did not have toilet facilities in their household, and 248 (74.00%) of the participants had ordinary (latrine) type of toilet. Similarly, 146 (43.60%) of the participants did not have handwashing facilities inside their toilets. A total of 205 (61.20%) participants had their private water source, and the majority (52.80%) used LPG gas as their primary fuel for daily cooking purposes (Table 1).

### 3.2. Knowledge of Food Safety

Table 2 below illustrates the participants' knowledge of food safety. Most of the food handlers (94.60%) knew that washing hands thoroughly before and after preparing food could prevent food contamination. In comparison, 62.10% of food handlers knew that hands should be washed during food preparation if the food handler touches their face, hair, and so forth. Moreover, 60.0% of food handlers agreed that using gloves during food handling reduces the risk of contamination, while 57.60% of food handlers said that wiping cloths used in the kitchen could spread microorganisms. However, only 20.30% of food handlers knew that vegetables and fruits could not be made germ-free by washing only with fresh water. Most of the food handlers (93.70%) knew that insects like cockroaches and flies can transfer foodborne pathogens; nevertheless, only 22.70% of food handlers knew that the same cutting board/knife could not be used for raw and cooked food, though that may look clean. The majority of the food handlers (78.50%) agreed that separating vegetables from animal products is an important step to prevent the transmission of germs, and 87.50% of food handlers agreed that raw food needs to be stored separately from cooked foods. Surprisingly, only 42.10% of food handlers knew that cooked foods should be thoroughly reheated, and only 7.80% knew that contaminated food may not have changed color, odor, or taste. Also, only 34.00% of food handlers knew that leftover food may be unsafe. It was found that 35.50% of food handlers knew that refrigerating food only slows bacterial growth, while 67.20% of food handlers knew that hot cooked food is good before serving. In addition to this, 62.70% of food handlers were aware that the purity of water cannot be known by the way it looks, and 74.90% of food handlers argued that foods should be from well-known sources and with a label identification.

### 3.3. Practices of Food Safety

Table 3 below shows the practices of the participants toward food safety. The majority, 89.00% of food handlers, washed their hands always before and after cooking. Only 51.90% of food handlers always cleaned surfaces and equipment used in food preparation before using them for the next food. Surprisingly, 51.60% of food handlers never washed their hands after touching their face, hair, rings, and so forth, during food preparation. Furthermore, 42.70% of food handlers do not taste food with unprotected hands. Only 29.30% of food handlers used distinct utensils and cutting boards for preparing raw and cooked food. Also, 42.70% of food handlers do not wash raw meat with water before preparing it, and nearly half (49.30%) store raw and cooked foods separately. In addition to this, 34.90% of food handlers reheat cooked food, while only 3.60% of the respondents never ate food kept at room temperature for over 2 h. About half of the respondents (50.40%) kept the leftover food at room temperature until the next meal sometimes. Furthermore, 62.10% of food handlers never verified the conditions of use and storage for prepackaged food, and 52.8% never read labels or expiry dates of food before buying or eating. At last, more than half of the food handlers (54.3%) always removed the moldy part from vegetables/fruits and ate the healthy parts of the same vegetables/fruits, and 40.3% of the respondents always ate fast foods like *chatpate*, *samosa, and pakora* wrapped in newspaper.

### 3.4. Level of Knowledge and Practices

**Figure 1:**
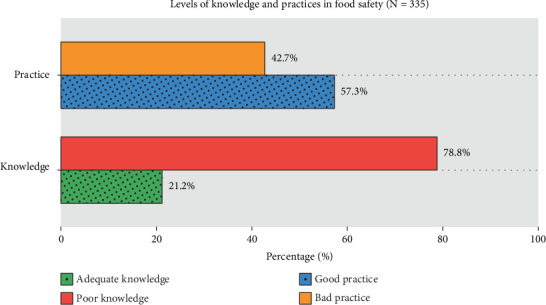
Level of knowledge and self-reported practices of the food handlers.

Figure 1 shows food handlers' overall knowledge and self-reported practices. The cutoff score was 12 for knowledge of food safety. Out of 335 food handlers, only 71 (21.20%) had adequate knowledge, while 264 (78.80%) had poor knowledge of food safety. Similarly, the cutoff score for the practices was 28, and more than half, 192 (57.30%) of food handlers had good food safety practices, and 143 (42.70%) had bad practices.

### 3.5. Factors Associated With Food Safety Practices

Factors associated with the self-reported practices were identified using binary logistic regression, presented in Table 4. Altogether, seven variables were significantly associated with the practices. The Omnibus Chi-square test (*χ*^2^ − statistic = 105.48, *p* value < 0.001) shows that the estimated model is statistically significant. According to the Hosmer–Lemeshow Chi-square test (*χ*^2^ − statistic = 10.95, *p* value = 0.204), the regression model is fitted well. A 41% variation of the outcome variable (practices of food safety) has been explained by the variations of independent variables, as shown by the Nagalkerke *R* square (0.41).

Education is one of the significant factors associated with food safety practices. People with no education were almost three times more likely to have bad food safety practices (AOR = 3.01, 95% CI [0.84–11.15]), and those who had received primary education were 2.5 times more likely to have bad practices (AOR = 2.50, 95% CI [1.20–5.31]) when compared to food handlers who had education attained above the primary level. On analyzing ethnic groups, food handlers from Muslim/Dalit/Madhesi groups collectively have bad food safety practices when compared to Brahmin/Chhetri by 2.43 times more (AOR = 2.43, 95% CI [1.07–5.57]). Similarly, respondents who had not heard/known about food safety were 2.48 times more likely to have bad food safety practices as expected (AOR = 2.48, 95% CI [1.27–4.90]). Regarding environmental factors, food handlers who do not have their water source were 1.88 times more likely to have bad practices (AOR = 1.88, 95% CI [0.95–3.76]). However, surprisingly, the food handlers who cook food using wood as fuel were 75% more likely to have good practices of food safety compared to those who used LPG (AOR = 0.25, 95% CI [0.08–0.72]). Moreover, food handlers who did not have a water facility inside the toilet were 1.94 times more likely to have bad practices of food safety (AOR = 1.94, 95% CI [0.91–4.21]) as compared to those who had such facilities. Lastly, the food handlers having poor knowledge of food safety were 4.40 times more likely to have bad practices of food safety, as expected (AOR = 4.40, 95% CI [1.82–12.08]).

## 4. Discussions

Food safety is very important as it is a basic need for human survival. Unhygienic foods are one of the major reasons for foodborne illness, which disturbs the daily life of everybody [31]. Hence, household food handlers need to be aware of proper food handling to control pathogen contamination. Also, their positive motivation will rely on the knowledge acquired by them, as enforceable guidelines do not regulate household food handling [32].

Out of 335 food handlers surveyed in this study, 57.30% had good practices of food safety. This is lower than studies done among household food handlers in Bangladesh [33], Lebanon [26], and India [34] but higher than food handlers in Ethiopia [2, 24, 35] and Pakistan [14]. This discrepancy might be due to differences in the food handlers' sociocultural, economic, and demographic characteristics and methodological differences.

The logistic regression found that the education level, ethnicity, previous awareness about food safety, water source ownership, fuel for cooking, water facility inside the toilet, and knowledge of food safety are the significant factors associated with the practices of food handlers.

Various studies have shown that educated food handlers have good practices in ensuring food safety in households, showing that education is a vital factor in ensuring food safety [15, 24]. Thus, a lack of knowledge on food handling issues could be a barrier to food handlers' practice of food safety [17, 35–37]. As educational attainment increases, the ability to understand the challenges and risks of having contaminated food also increases. So, as expected, the educated food handlers have shown good practices for assuring food safety as per the findings of this study.

In Nepal, ethnic groups like Madheshi, Dalit, and Muslim are economically backward compared to the higher castes [38]. Since health and awareness align with socioeconomic status, the mode of food preparation, handling, and consumption also varies. As per the findings, the food handlers belonging to socially and economically backward ethnic groups do not have good practices compared to higher castes.

Safe food handling practices are more common in economically sound households. Due to the lack of improved water supply and sanitary facilities, food safety problems can be exacerbated in low socioeconomic settings. Also, water is an essential component in the food safety chain; as besides being used in cleaning food products, it also forms a part of our food [39]. Thus, a safe and easily available water facility is directly linked with good food handling practices, consequently important for public health, according to the findings of this study. Food handlers who do not have a private water source and no water facilities inside the toilets do not have good practices. Similar studies from Kenya and Ethiopia support this conclusion [24, 40]. Thus, the findings imply water is a fundamental necessity for maintaining better personal hygiene, which in turn helps to reduce the potential risks of food contamination and diseases.

Environmental hygiene is important for food safety and necessary to support safe food handling by food handlers. The study by [40] found a higher occurrence of food contamination among those who did not have access to running water near the toilet facilities compared to those who did. This implied the importance of water in the sanitation processes. Access to a toilet facility and the availability of running water within the toilet facility were the environmental factors found likely to decrease the risk of food contamination. Since water availability is directly associated with the maintenance of hygiene in and out of the kitchen, food handlers who have public water sources do not have easy access to water, resulting in compromised sanitation. As a result, food safety in such households is compromised.

## 5. Conclusions

In conclusion, the study done among the household food handlers in the Budhiganga Rural Municipality of Nepal found an average level of safe food practices. The logistic regression analysis identified education level, ethnicity, prior awareness of food safety, ownership of water source, type of cooking fuel, availability of water within toilet facilities, and knowledge of food safety as significant predictors of food safety practice. Since household food handlers are at the ultimate stage of defending against contamination in the food safety chain, educating and creating food safety awareness should cover all the food handlers who are not engaged in educational institutions and are limited to their households. Similarly, strengthening the families' socioeconomic conditions could significantly contribute to safe food practices. Though most Nepalese have their meals daily at home, there are negligible studies done regarding food safety in the household. Researchers and officials in relevant government agencies should be aware that tremendous legislative, agricultural, industrial, and public health efforts are needed to improve the safety of the food supply. But these efforts are only worthwhile if matched by safe food handling at home.

## Figures and Tables

**Table 1:** Sociodemographic characteristics of the participants.

**Variables**	**Categories**	**Frequency (** **n** **)**	**Percent (%)**
Age	≤ 24 years	29	8.7
	25–44 years	223	66.6
	≥ 45 years	83	24.8

Education	No education	52	15.5
	Primary education	145	43.3
	Secondary or above education	138	41.2

Ethnicity	Janajati	80	23.9
	Brahmin/Chhetri	141	42.1
	Madheshi/Dalit/Muslim	114	34.0

Income	Less than NRs. 20,000	143	42.7
	NRs. 20,000–30,000	106	31.6
	More than NRs. 30,000	86	25.7

Number of family members	≤ 5	247	73.7
	> 5	88	26.3

Presence of children (less than 15 years) in the household	No	76	22.7
	Yes	259	77.3

Heard/know about food safety	No	199	59.40
	Yes	136	40.60

Toilet facility	No	39	11.6
	Yes	296	88.4

Type of toilet	Modern (with flush system)	48	14.3
	Ordinary (latrine)	248	74.0
	No toilet facilities	39	11.6

Hand washing facility inside the toilet	No	146	43.6
	Yes	150	44.8
	No toilet facilities	39	11.6

Water source	Private	205	61.2
	Public	130	38.8

Fuel for cooking	LPG gas	177	52.8
	Biogas	32	9.6
	Wood	58	17.3
	Others (cow dung cakes, rice husk, etc.)	68	20.3

*Note: *1 USD = NRs.132.60 during the study time.

**Table 2:** Knowledge of food safety among food handlers in the households of Budhiganga Rural Municipality.

**Statements**	**Response, ** **n** ** (%)**		
	**Yes**	**No**	**I do not know**
Hands must be thoroughly washed before and after preparing food to prevent contamination.	317 (94.60)	5 (1.50)	13 (3.90)
During food preparation, hands should be washed after touching the face, hair, etc.	208 (62.10)	112 (33.40)	15 (4.50)
The use of gloves while handling food reduces the risk of food contamination.	201 (60.00)	76 (22.70)	58 (17.30)
Wiping cloths can spread microorganisms.	193 (57.60)	62 (18.50)	80 (23.90)
Vegetables and fruits can be made germ-free by using only drinking water.	248 (74.00)	68 (20.30)	19 (5.70)
Insects like cockroaches and flies can transfer foodborne pathogens.	314 (93.70)	8 (2.40)	13 (3.90)
The same cutting board/knife can be used for raw and cooked foods, provided it looks clean.	252 (75.20)	76 (22.70)	7 (2.10)
Separating vegetables from animal products is important to prevent the transmission of germs.	263 (78.50)	18 (5.40)	54 (16.10)
Raw food needs to be stored separately from cooked food.	293 (87.50)	21 (6.30)	21 (6.30)
Cooked foods do not need to be thoroughly reheated.	191 (57.00)	141 (42.10)	3 (0.90)
Contaminated foods always have some change in color, odor, or taste.	280 (83.60)	26 (7.80)	29 (8.70)
Leftover food that smells good is still safe to eat.	215 (64.20)	114 (34.00)	6 (1.80)
Refrigerating food only slows bacterial growth.	119 (35.50)	66 (19.70)	150 (44.80)
Cooked food should be kept very hot before serving.	225 (67.20)	107 (31.90)	3 (0.90)
Safe water can be identified by the way it looks.	119 (35.50)	210 (62.70)	6 (1.80)
Food must be from reliable sources and identified with a label.	251 (74.90)	14 (4.20)	70 (20.90)

**Table 3:** Self-reported practices of food handlers in the households of Budhiganga Rural Municipality.

**Statements**	**Response, ** **n** ** (%)**		
	**Always**	**Sometimes**	**Never**
Do you wash your hands before and after cooking?	298 (89.00)	37 (11.04)	0 (0.00)
Do you clean surfaces and equipment used for food preparation before reusing other food?	174 (51.90)	140 (41.80)	21 (6.30)
Do you wash your hands after touching your face, hair, rings, etc., during food preparation?	37 (11.00)	125 (37.30)	173 (51.60)
Do you taste food with unprotected hands?	83 (24.80)	109 (32.50)	143 (42.70)
Do you use separate utensils and cutting boards when preparing raw and cooked food?	98 (29.30)	191 (57.00)	46 (13.70)
Do you wash raw meat with running water before cutting or preparing it?	59 (17.60)	133 (39.70)	143 (42.70)
Do you store raw and cooked foods separately?	165 (49.30)	143 (42.70)	27 (8.10)
Do you reheat cooked food until it is hot throughout?	117 (34.90)	178 (53.10)	40 (11.90)
Do you consume food kept at room temperature (more than 2 h)?	159 (47.50)	164 (49.00)	12 (3.60)
Do you keep the leftover food at room temperature until the next meal?	116 (34.60)	169 (50.40)	50 (14.90)
Do you check the conditions of use and storage of prepackaged food?	48 (14.30)	79 (23.60)	208 (62.10)
Do you read labels or the expiry date of packaged food before purchasing or eating?	44 (13.10)	114 (34.00)	177 (52.80)
Do you remove the moldy part from vegetables/fruits and eat the healthy parts of the same vegetables/fruits?	182 (54.30)	147 (43.90)	6 (1.80)
Do you eat fast foods (*chatpate*, *samosa*, *pakoda*, etc.) wrapped in a newspaper?	135 (40.30)	158 (47.20)	42 (12.50)

**Table 4:** Results of bivariate and multiple binary logistic regression models of factors associated with self-reported food safety practices.

**Variables**	**Categories**	**Good practices**	**Bad practices**	**COR (95% CI)**	**AOR (95% CI)**
		**Frequency (%)**	**Frequency (%)**		
Age	≤ 24 years^a^	16 (64)	9 (36)	1	
	25–44 years	130 (66.67)	65 (33.33)	0.89 (0.38–2.2)	0.85 (0.26–2.80)
	≥ 45 years	45 (59.21)	31 (40.79)	1.22 (0.49–3.22)	0.60 (0.16–2.26)

Education	No education	11 (40.74)	16 (59.26)	6.91 (2.89–17.17)	3.01 (0.84–11.15)⁣^∗^
	Primary education	66 (50.38)	65 (49.62)	4.68 (2.71–8.29)	2.50 (1.20–5.31)⁣^∗∗^
	Secondary or above education^a^	114 (82.61)	24 (17.39)	1	

Ethnicity	Madhesi/Dalit/Muslim	32 (39.51)	49 (60.49)	4.3 (2.42–7.78)	2.43 (1.07–5.57)⁣^∗∗^
	Janajati	55 (74.32)	19 (25.68)	0.97 (0.5–1.83)	0.94 (0.40–2.20)
	Brahmin/Chhetri^a^	104 (73.76)	37 (26.24)	1	

Income	< NRs. 20,000	60 (60.61)	39 (39.39)	3.59 (1.89–7.1)	1.31 (0.46–3.71)
	NRs. 20,000–30,000	61 (54.95)	50 (45.05)	2.84 (1.47–5.71)	1.70 (0.71–4.19)
	> NRs. 30,000^a^	70 (81.4)	16 (18.6)	1	—

Family members	≤ 5^a^	162 (69.23)	72 (30.77)	1	—
	> 5	29 (46.77)	33 (53.23)	2.56 (1.45–4.55)	1.12 (0.53–2.37)

Presence of children (less than 15 years) in the household	Yes	144 (64.57)	79 (35.43)	0.99 (0.57–1.74)	1.11 (0.52–2.40)
	No^a^	47 (64.38)	26 (35.62)	1	

Heard/know about food safety	Yes^a^	109 (80.15)	27 (19.85)	1	
	No	82 (51.25)	78 (48.75)	3.84 (2.3–6.56)	2.48 (1.27–4.90)⁣^∗∗^

Water source	Private^a^	152 (74.15)	53 (25.85)	1	
	Public	39 (42.86)	52 (57.14)	3.82 (2.28–6.47)	1.88 (0.95–3.76)⁣^∗^

Fuel for cooking	LPG^a^	126 (71.19)	51 (28.81)	1	
	Biogas	11 (45.83)	13 (54.17)	2.92 (1.23–7.07)	1.56 (0.48–5.09)
	Wood	37 (66.07)	19 (33.93)	1.27 (0.66–2.39)	0.25 (0.08–0.72)⁣^∗∗^
	Others	17 (43.59)	22 (56.41)	3.2 (1.58–6.59)	0.73 (0.23–2.30)

Type of toilet	Modern^a^	43 (89.58)	5 (10.42)	1	
	Ordinary	148 (59.68)	100 (40.32)	5.81 (2.43–17.24)	2.17 (0.66–8.19)

Hand washing facility inside the toilet	Yes^a^	73 (50)	73 (50)	1	
	No	118 (78.67)	32 (21.33)	3.69 (2.24–6.19)	1.94 (0.91–4.21)⁣^∗^

Knowledge	Adequate^a^	64 (90.14)	7 (9.86)	1	
	Poor	127 (56.44)	98 (43.56)	7.06 (3.3–17.52)	4.40 (1.82–12.08)⁣^∗∗^

Intercept					0.01 (0.00–0.08)⁣^∗∗^

^a^Reference category.

⁣^∗^Significant at a 10% level of significance and ⁣^∗∗^significant at a 5% level of significance.

## Data Availability

The data used in the study are available upon request to the corresponding author.

## Nomenclature

AOR: adjusted odds ratio
CI: confidence interval
COR: crude odds ratio
WHO: World Health Organization
